# Composition-Dependent Lithographic Performance of 3-Arm Star Photoresists

**DOI:** 10.3390/polym18212612

**Published:** 2026-10-22

**Authors:** Yura Choi, Jinyoung Kim, Namchul Cho

**Affiliations:** Department of Energy Engineering, Soonchunhyang University, 22 Soonchunhyang-ro, Asan 31538, Republic of Korea; bnbn3237@gmail.com (Y.C.); thn05007@gmail.com (J.K.)

**Keywords:** 3-arm star polymer, RAFT polymerization, copolymer composition, chemically amplified resist, negative-tone imaging, post-exposure baking

## Abstract

The lithographic performance of chemically amplified resists is strongly influenced by the balance among the functional components of the resist polymer. In this study, a series of 3-arm star terpolymers containing 2-hydroxyethyl methacrylate (HEMA), tetrahydrodicyclopentadienyl methacrylate (TCDMA), and the acid-labile monomer 2-methyl-2-adamantyl methacrylate (MAMA) were synthesized via core-first reversible addition–fragmentation chain-transfer (RAFT) polymerization using a trifunctional TMP-CEPA3 chain-transfer agent. Three polymers, denoted HTM 442, HTM 334, and HTM 226, were prepared by systematically varying the MAMA fraction. ^1^H NMR analysis confirmed different incorporated monomer compositions, while GPC analysis showed relatively narrow molecular-weight distributions for all polymers. Thermal analysis revealed that the thermophysical properties were governed by the combined effects of HEMA, TCDMA, and MAMA rather than by the MAMA fraction alone. Negative-tone lithographic evaluation using 365 nm UV exposure and *n*-butyl acetate development demonstrated composition-dependent differences in pattern formation. Among the investigated compositions, HTM 334 exhibited the most favorable line-pattern morphology, with relatively uniform line profiles and lower LER values at most of the investigated nominal mask dimensions. HTM 442 also formed distinguishable patterns, whereas HTM 226 did not produce discernible patterns under the evaluated conditions. These results indicate that increasing the acid-labile MAMA fraction does not lead to a monotonic improvement in lithographic performance within the investigated composition range, highlighting the need to consider the overall copolymer composition when designing 3-arm star photoresists.

## 1. Introduction

Chemically amplified resists (CARs) have played a central role in high-resolution photolithography because a single photochemical event can initiate multiple acid-catalyzed reactions during post-exposure baking, thereby providing high imaging sensitivity [1,2,3,4,5]. Recent studies have further demonstrated that the photosensitivity and lithographic response of polymeric resists can be effectively tuned through functional-group engineering and optimization of acid-labile protecting groups [6,7]. In negative-tone imaging (NTI) using organic-solvent developers, exposure-generated acid and subsequent post-exposure baking induce chemical changes in the resist polymer that alter its polarity and developer affinity, allowing the unexposed regions to be preferentially removed while the exposed regions remain [8,9]. Consequently, lithographic performance is strongly affected by the chemical structure of the polymer matrix, including its functional-group composition, molecular architecture, thermal properties, and interaction with the organic-solvent developer [1,2,10,11,12]. Designing an appropriate polymer structure is therefore essential for generating sufficient dissolution contrast between the unexposed and exposed regions during organic-solvent development [13,14].

Methacrylate-based photoresists frequently incorporate multiple functional monomers to satisfy the different chemical and physical requirements of the lithographic process. Polar functionalities can influence polymer–developer interactions, whereas bulky cycloaliphatic groups are commonly introduced to provide hydrophobicity, rigidity, and resistance to the processing environment [2,15,16,17]. Acid-labile adamantyl-containing methacrylates are particularly attractive because acid-catalyzed deprotection induces a substantial change in polymer polarity and, consequently, in polymer–developer interactions [15,16,18,19,20]. In organic-solvent-based negative-tone development, this polarity change can reduce the solubility of the exposed region in the developer, thereby generating the dissolution contrast required for negative-tone pattern formation.

Adamantyl-containing methacrylates have been investigated as resist components because their bulky cycloaliphatic structures and acid-responsive functionalities can strongly affect thermal properties, polarity, and dissolution behavior. Barkakaty et al. reported acid-labile adamantyl methacrylate monomers containing hemiacetal functionalities and demonstrated the importance of the protecting-group structure for acid sensitivity and thermal behavior [20]. More recently, Kim et al. showed that incorporation of adamantyl groups into (meth)acrylate photoresist polymers substantially modifies polymer dissolution behavior in aqueous TMAH developer [18]. In addition, recent studies have emphasized that functional-group polarity, hydrophilicity, and the chemical environment surrounding acid-responsive groups can significantly influence resist sensitivity and pattern fidelity [7,14,21,22]. These studies establish the importance of adamantyl and polarity control in photoresist design; however, they do not directly address how the fraction of an acid-labile adamantyl monomer affects organic-solvent negative-tone pattern formation within a fixed multiarm polymer architecture.

However, increasing the protecting-group fraction does not necessarily improve every aspect of resist performance. Changes in the protected-monomer fraction simultaneously alter the overall hydrophobicity, chain packing, thermal behavior, deprotection environment, and polymer–developer interactions [21,22,23]. Thus, lithographic behavior should be considered in terms of the overall balance among the functional monomer units rather than the acid-labile component alone. Importantly, previous studies have not directly addressed how variation in the fraction of an acid-labile adamantyl monomer influences organic-solvent negative-tone pattern formation within a fixed multiarm polymer architecture.

In addition to chemical composition, macromolecular architecture provides another means of tuning photoresist behavior. Branched and star-shaped polymers have attracted interest as resist matrices because their compact architectures can differ substantially from conventional linear polymers in chain dimensions, packing, and dissolution behavior [24,25,26,27]. RAFT polymerization has previously been employed to prepare multicomponent photoresist polymers and star-shaped chemically amplified resists. Li et al. demonstrated MAMA-containing multicomponent RAFT polymers for KrF lithography [24], whereas Zheng et al. reported RAFT-derived star polymers as chemically amplified positive-tone photoresists [25]. More recent studies have further extended controlled and multiarm polymer architectures for photoimaging applications [26,28]. These studies demonstrate the utility of RAFT and star architectures in resist design, but systematic evaluation of incorporated functional-monomer composition within the same star-polymer platform under organic-solvent negative-tone development remains limited. Nevertheless, controlling the actual composition of multicomponent star copolymers remains important when monomers with substantially different structures are copolymerized. In particular, HEMA, TCDMA, and MAMA differ considerably in polarity and steric environment, and the nominal monomer feed composition may therefore not be directly reproduced in the final copolymer composition. Such deviations can complicate the interpretation of structure–property relationships because lithographic behavior must ultimately be correlated with the incorporated polymer composition rather than with the nominal feed ratio [29,30]. Although star-shaped photoresist polymers and composition-controlled methacrylate resists have been investigated separately, the influence of the actual incorporated monomer composition on negative-tone pattern formation in multicomponent star-shaped resist systems remains less systematically understood. In particular, how the balance among polar, cycloaliphatic, and acid-labile functionalities affects organic-solvent development and PEB-dependent patterning behavior requires further clarification.

In this study, we prepared a series of acid-stable 3-arm star terpolymers via core-first RAFT polymerization using the trifunctional TMP-CEPA3 chain-transfer agent. Three polymers, designated HTM 442, HTM 334, and HTM 226, were synthesized using HEMA:TCDMA:MAMA feed ratios of 40:40:20, 30:30:40, and 20:20:60, respectively. Thus, the nominal MAMA feed fraction was varied from 20 to 60 mol% while maintaining an HEMA:TCDMA feed ratio of 1:1. The actual incorporated monomer compositions were subsequently determined by ^1^H-NMR spectroscopy, enabling the lithographic behavior to be evaluated in relation to the experimentally determined copolymer composition rather than the nominal feed ratio alone. The resulting polymers were characterized in terms of chemical composition, molecular-weight distribution, and thermophysical properties, followed by evaluation of their exposure-responsive behavior and lithographic pattern formation. By comparing polymers with systematically different functional-monomer compositions within the same 3-arm star platform, this study aims to clarify whether lithographic performance is governed primarily by the absolute fraction of the acid-labile MAMA unit or by the overall balance among the polar, cycloaliphatic, and acid-responsive functionalities. The three feed compositions were intentionally selected to represent low, intermediate, and high MAMA fractions at nominal values of 20, 40, and 60 mol%, respectively, while maintaining a constant HEMA:TCDMA feed ratio of 1:1. This three-point design was intended to examine whether increasing the acid-labile MAMA fraction produces a monotonic change in lithographic behavior within the same 3-arm star-polymer platform, rather than to map the complete ternary composition space.

## 2. Materials and Methods

### 2.1. Materials

The base methacrylate monomers, including 2-hydroxyethyl methacrylate (HEMA) and tetrahydrodicyclopentadienyl methacrylate (TCDMA), were purchased from Tokyo Chemical Industry (Tokyo, Japan), and 2-methyl-2-adamantyl methacrylate (MAMA) was purchased from Sigma-Aldrich (St. Louis, MO, USA). Trimethylolpropane (TMP) was purchased from Sigma-Aldrich (St. Louis, MO, USA). N-(trifluoromethylsulfonyloxy)-5-norbornene-2,3-dicarboximide (NDI), used as the photoacid generator (PAG) in the chemically amplified photoresist formulations, was purchased from Burke Gilman Co., Ltd. (Asan, Republic of Korea).

The trithiocarbonate-based RAFT precursor, 4-cyano-4-[(dodecylsulfanylthiocarbonyl)sulfanyl]pentanoic acid (CEPA), was used to synthesize the acid-stable 3-arm chain transfer agent. CEPA was purchased from Sigma-Aldrich (St. Louis, MO, USA).

N,N′-dicyclohexylcarbodiimide (DCC) and 4-dimethylaminopyridine (DMAP) were purchased from Sigma-Aldrich (St. Louis, MO, USA). These materials were employed as the coupling agent and catalyst, respectively.

2,2′-Azobis(2,4-dimethylvaleronitrile) (V-65) was purchased from FUJIFILM Wako Pure Chemical Corporation (Osaka, Japan). Propylene glycol monomethyl ether acetate (PGMEA) was purchased from Sigma-Aldrich (St. Louis, MO, USA). N-((trifluoromethylsulfonyl)oxy)-5-norbornene-2,3-dicarboximide (NDI) was used as the photoacid generator (PAG) for the chemically amplified photoresist formulations. Propylene glycol monomethyl ether acetate (PGMEA) and diethylene glycol monoethyl ether (DGME) were used as formulation solvents. *n*-Butyl acetate (*n*-BA) was used as the organic developer for negative-tone imaging, and *n*-hexane was used as the rinsing solvent after development. These solvents were purchased from DUKSAN (Seoul, Republic of Korea). All reagents and solvents were used without further purification unless otherwise specified.

### 2.2. Synthesis of the 3-Arm Chain-Transfer Agent (TMP-CEPA3)

The 3-arm chain-transfer agent (TMP-CEPA3) was synthesized via an esterification reaction between trimethylolpropane (TMP) and the carboxylic-acid-functionalized RAFT precursor, 4-cyano-4-[(dodecylsulfanylthiocarbonyl)sulfanyl]pentanoic acid (CEPA), using N,N′-dicyclohexylcarbodiimide (DCC) as the coupling agent and 4-dimethylaminopyridine (DMAP) as the catalyst. TMP (0.40 g, 2.98 mmol), CEPA (2.82 g, 6.99 mmol), and DMAP (0.11 g, 0.89 mmol) were introduced into a dried jacketed reactor. The reactor was subjected to three consecutive vacuum–nitrogen cycles to establish an inert and anhydrous atmosphere and was subsequently cooled to 0 °C.

Anhydrous tetrahydrofuran (THF, 100 mL) was added to the reactor, and the reaction mixture was stirred for 30 min at 0 °C. Separately, DCC (2.76 g, 13.42 mmol) was dissolved in dichloromethane (DCM, 20 mL), and the resulting solution was added dropwise to the reaction mixture at 0 °C. During the addition, formation of a white precipitate of dicyclohexylurea (DCU) was observed. After completion of the DCC addition, the reaction mixture was gradually warmed to 25 °C and stirred for 48 h.

After the reaction, the precipitated DCU was removed by filtration, and THF and DCM were removed under reduced pressure. The resulting crude product was redissolved in DCM and sequentially washed with 0.5 N aqueous HCl and saturated aqueous NaHCO3 solutions to remove residual basic species and other water-soluble impurities. The organic phase was collected, and DCM was removed under reduced pressure.

The crude product was subsequently purified by silica-gel column chromatography using ethyl acetate/*n*-hexane (1:8, *v*/*v*) as the eluent. After removal of the solvent under reduced pressure, the purified trifunctional RAFT chain-transfer agent, TMP-CEPA3, was obtained.

### 2.3. Synthesis of 3-Arm Star Terpolymers (HTM Polymers)

Three 3-arm star terpolymers with different nominal HEMA:TCDMA:MAMA feed ratios were synthesized via core-first RAFT polymerization using TMP-CEPA3 as the multifunctional chain-transfer agent. The polymers were designated HTM 442, HTM 334, and HTM 226 according to nominal HEMA:TCDMA:MAMA feed ratios of 40:40:20, 30:30:40, and 20:20:60, respectively.

In a representative polymerization procedure, the predetermined amounts of HEMA, TCDMA, and MAMA were introduced into a dry jacketed reactor together with PGMEA and stirred to obtain a homogeneous monomer solution. Separately, V-65 and TMP-CEPA3 were dissolved in PGMEA, and the resulting initiator/CTA solution was added to the monomer mixture. The reactor was purged with nitrogen to establish an inert atmosphere prior to polymerization.

The reaction mixture was heated to 55 °C and stirred for 20 h. After polymerization, the reaction was quenched by cooling the reactor in an ice-water bath. The quenched polymer solution was diluted with THF and concentrated under reduced pressure. The resulting polymer was redissolved in THF, and the solution was added dropwise into approximately eight volumes of heptane to precipitate the polymer. The precipitated polymer was collected by filtration and dried under vacuum at 40 °C.

The same polymerization and purification procedure was applied to all three nominal feed compositions. The incorporated HEMA, TCDMA, and MAMA ratios in the isolated polymers were subsequently determined by ^1^H NMR spectroscopy rather than assumed from the nominal monomer feed ratios.

### 2.4. Characterization

Proton nuclear magnetic resonance (^1^H NMR) measurements were performed using a 400 MHz Ascend 400 spectrometer (Bruker Corporation, Billerica, MA, USA) with CDCl_3_ as the solvent and 64 scans. Molecular weight distributions were determined by gel permeation chromatography (GPC, Waters Corporation, Milford, MA, USA) using THF as the mobile phase. The mobile phase flow rate was set to 0.1 mL/min, and the GPC system was equipped with KS-801, KS-802, and KS-803 columns, along with a differential refractive index (RI 804) detector (Agilent Technologies, Santa Clara, CA, USA). Differential scanning calorimetry (DSC, Q20 from TA Instruments Ltd., New Castle, DE, USA) was performed under a nitrogen atmosphere using repurified polymers that had been vacuum-dried at 40 °C for more than 24 h prior to analysis. Each sample was equilibrated at 40 °C for 5 min, heated to 130 °C at a rate of 5 °C min^−1^, held at 130 °C for 2 min, air-cooled to approximately 40 °C, and subsequently subjected to a second heating cycle to 130 °C at the same heating rate. The upper temperature was limited to 130 °C to avoid entering the first major thermal mass-loss region identified by TGA. The first-stage weight-loss temperatures were determined from the initial major thermal event observed in the TGA curves (TGA Q 500, TA Instruments, Advanced Energy and Display Materials Analysis Center, NFEC-2026-03-313944, TA Instruments Ltd., New Castle, DE, USA) in the range of 30–400 °C at a heating rate of 10 °C per minute under a nitrogen atmosphere.

FT–IR spectra were recorded using an FT-200 spectrophotometer (Horiba, Kyoto, Japan). For semi-quantitative FT-IR analysis, the transmittance spectra were converted to absorbance, and the O–H and C=O regions at 3200–3600 and 1650–1800 cm^−1^, respectively, were integrated after identical baseline correction. The integrated O–H area was normalized to the integrated C=O area, and the relative attenuation was calculated as

(1) $$[1-\frac{{{(A}_{O-H}\;/\;A_{C=O})}_{\mathrm{after}}}{{{(A}_{O-H}\;/\;A_{C=O})}_{\mathrm{before}}}]\times 100\;$$

Pattern images were captured using an optical microscope (BX53M, Olympus Corporation, Tokyo, Japan) at various magnifications. The experimental data were processed and plotted using Origin 2018 (OriginLab Corporation, Northampton, MA, USA).

### 2.5. Pattern Formation and Evaluation

Photoresist solutions were prepared by dissolving 0.5 g of each synthesized 3-arm star terpolymer (HTM 442, HTM 334, or HTM 226) and 25 mg of N-((trifluoromethylsulfonyl)oxy)-5-norbornene-2,3-dicarboximide (NDI) as the photoacid generator (PAG) in a mixed solvent of PGMEA and DGME at a volume ratio of 7:3. The mixtures were stirred for 4 h at room temperature to ensure complete dissolution.

The resulting photoresist solutions were spin-coated onto bare 1 × 1 cm silicon wafers at 1000 rpm for 1 min. The coated films were subsequently soft-baked on a hot plate at 80 °C for 1 min to remove residual solvent. After cooling, a photomask was aligned with each coated wafer, and the films were exposed to a 365 nm UV light source at a dose of 30 mJ cm^−2^. To investigate the effect of post-exposure baking temperature on pattern formation, the exposed films were subjected to PEB at 60, 70, 80, or 90 °C for 1 min. The wafers were then developed by immersion in *n*-butyl acetate for 10 s, followed by rinsing with *n*-hexane.

Finally, the patterned films were hard-baked at 80 °C for 1 min. The resulting negative-tone patterns were examined by optical microscopy to evaluate the influence of PEB temperature on pattern formation. The same photomask pattern was used for all formulations.

## 3. Results and Discussion

### 3.1. Compositional Tuning and Structural Characterization

**Figure 1:**
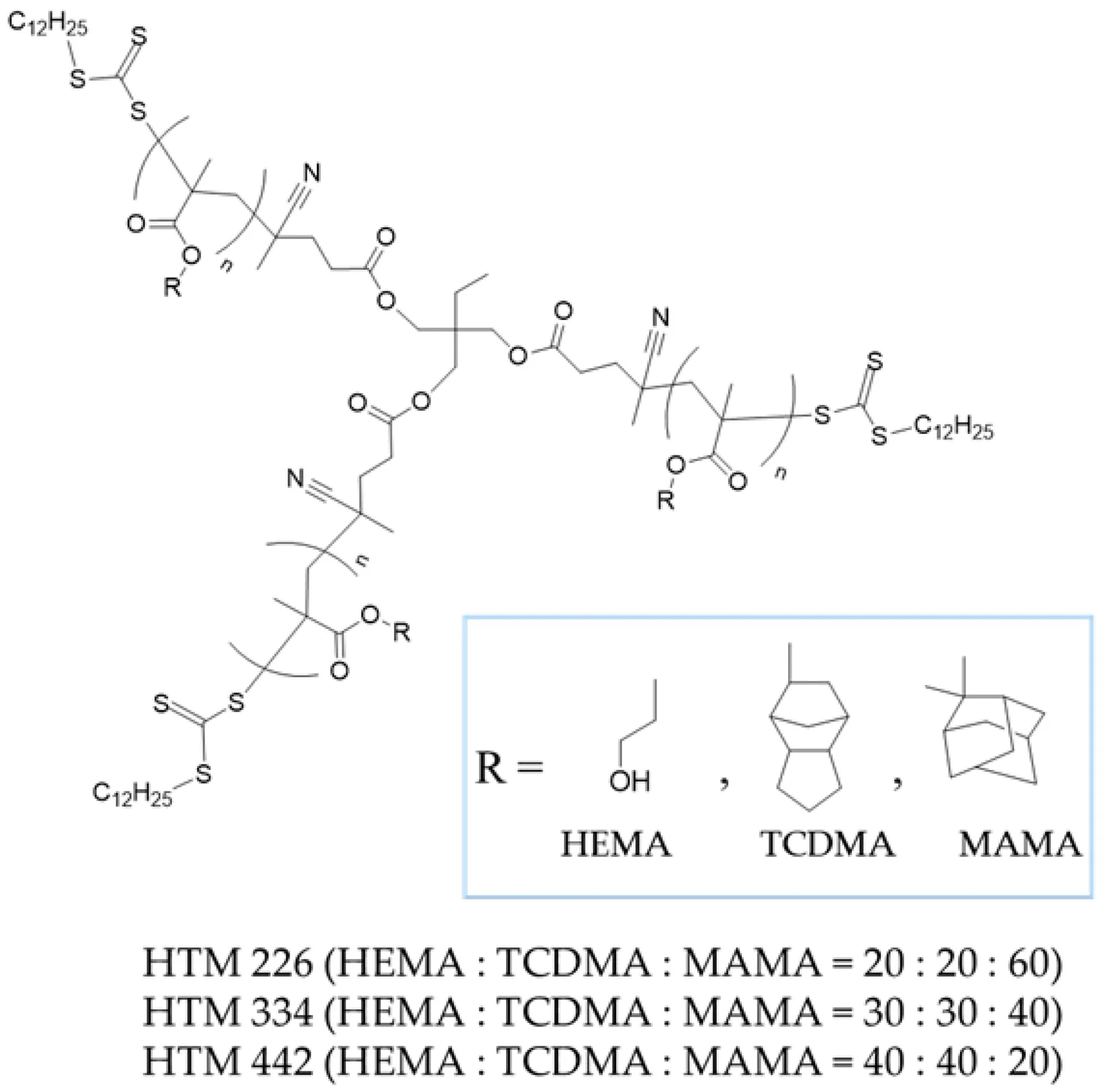
Schematic representation of the 3-arm star terpolymer synthesized via core-first RAFT polymerization and the chemical structures of HEMA, TCDMA, and MAMA.

To investigate the effect of copolymer composition on the properties of the 3-arm star photoresists, three terpolymers with different nominal monomer feed ratios were synthesized via core-first RAFT polymerization using the trifunctional TMP-CEPA3 chain-transfer agent. The resulting polymers were designated HTM 442, HTM 334, and HTM 226 according to the nominal HEMA:TCDMA feed ratios of 40:40:20, 30:30:40, and 20:20:60, respectively (Figure 1). In this series, the nominal MAMA feed fraction was varied from 20 to 60 mol%, while the HEMA feed ratio was maintained at 1:1. The sample codes therefore refer to the nominal monomer feed ratios rather than the final incorporated copolymer compositions.

**Figure 2:**
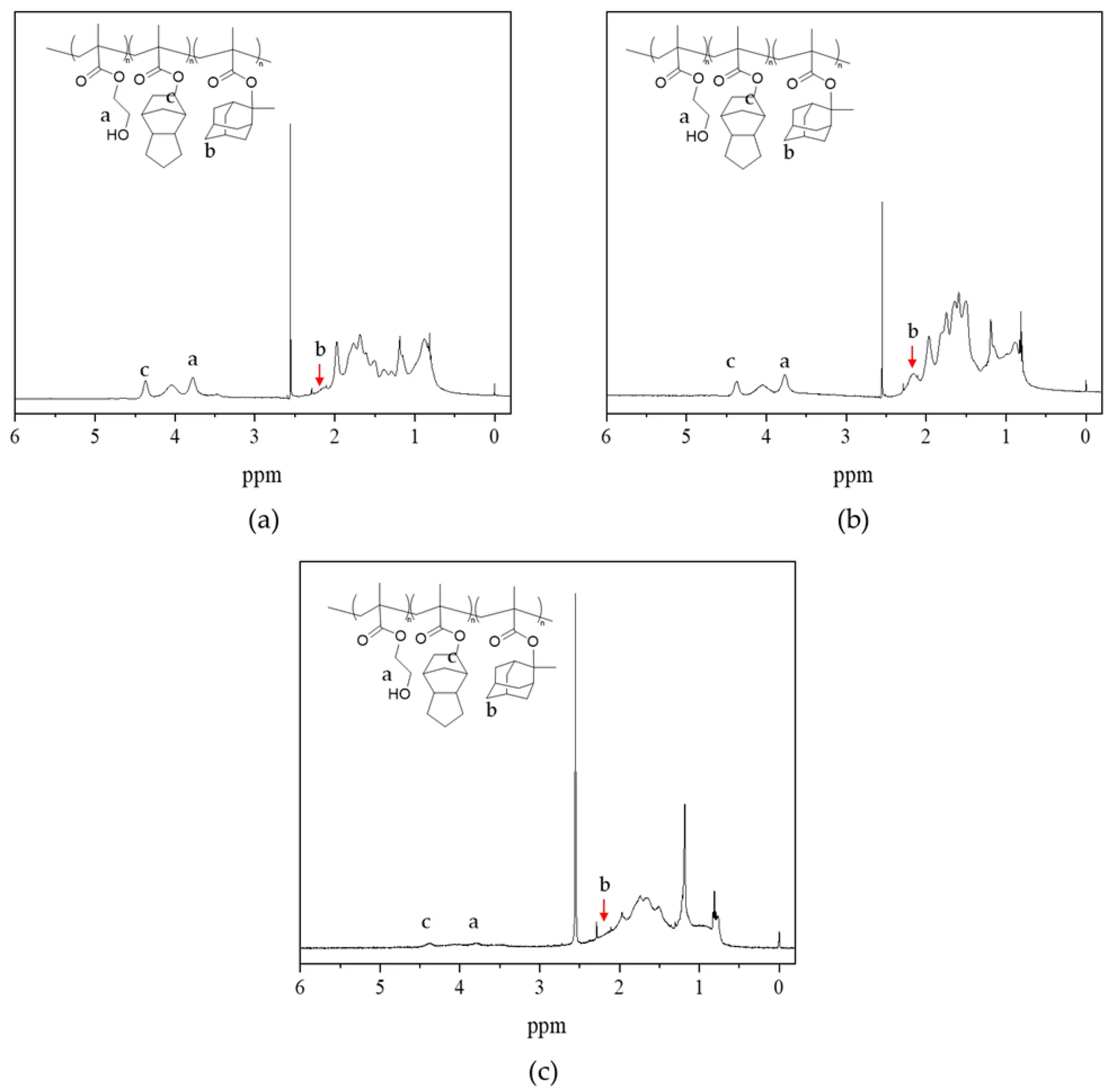
^1^H-NMR spectra of the synthesized 3-arm star terpolymers used to determine the incorporated monomer compositions: (**a**) HTM 442, (**b**) HTM 334, and (**c**) HTM 226. The copolymer compositions were determined from the integrated characteristic resonances (a–c) based on the overall spectral assignments rather than from a single isolated resonance.

The chemical structures and incorporated monomer compositions of the synthesized polymers were examined by ^1^H NMR spectroscopy, as shown in Figure 2. All three polymers exhibited characteristic proton resonances associated with the HEMA, TCDMA, and MAMA units, confirming the incorporation of the three functional methacrylate monomers into the polymer chains. The relative intensities of the characteristic signals varied among the three samples, reflecting differences in the incorporated monomer compositions.

Quantitative analysis of selected proton resonances was used to determine the relative molar fractions of the incorporated monomer units. As summarized in Table 1, the HEMA:TCDMA compositions determined by ^1^H NMR were approximately 3.0:4.4:3.1 for HTM 442, 3.0:3.3:4.0 for HTM 334, and 2.0:1.8:5.7 for HTM 226. These values correspond to MAMA fractions of approximately 30, 39, and 60 mol%, respectively. Thus, systematic variation in the nominal MAMA feed fraction produced a compositionally distinct series of star terpolymers, although the incorporated compositions did not exactly reproduce the nominal feed ratios.

The observed differences between the nominal feed ratios and the compositions of the isolated polymers may reflect non-equivalent monomer incorporation during polymerization and compositional fractionation during subsequent isolation and purification. Because conversion-dependent monomer consumption was not measured, the individual monomer conversions cannot be determined from the present data. HEMA, TCDMA, and MAMA differ in polarity and steric environment, and these structural differences may contribute to their different incorporation tendencies during RAFT polymerization. Accordingly, the experimentally determined copolymer compositions, rather than the nominal feed ratios alone, were used to interpret the subsequent composition-dependent properties of the synthesized photoresists. The reported compositions represent the isolated polymers and should not be interpreted as direct measurements of individual monomer conversion during polymerization.

To provide a quantitative description of the acid-responsive functionality, the average number of acid-labile MAMA units per star polymer was estimated from the experime ntally determined copolymer compositions together with the GPC molecular-weight data (Table 2). The estimated numbers of MAMA units were approximately 38, 59, and 62 for HTM 442, HTM 334, and HTM 226, respectively. These estimates provide a quantitative comparison of the relative acid-responsive functionality among the three polymer compositions and support the subsequent discussion of their lithographic behavior.

**Figure 3:**
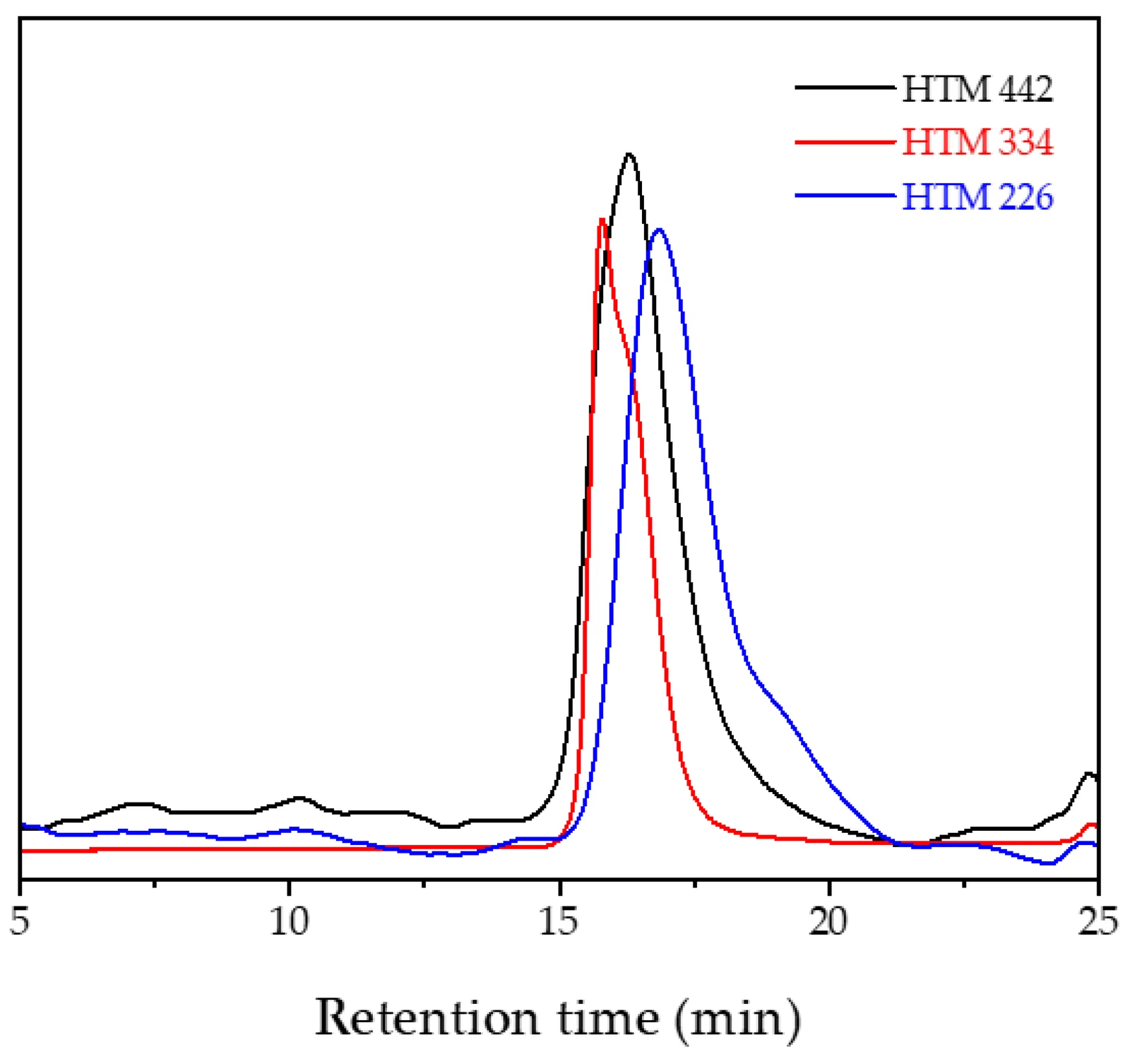
Gel permeation chromatography (GPC) traces of HTM 442, HTM 334, and HTM 226.

The molecular-weight characteristics of the three polymers were further examined by gel permeation chromatography (GPC), and the corresponding results are summarized in Table 1 and Figure 3. All three polymers exhibited dominant unimodal molecular-weight distributions with relatively narrow dispersities (Đ = 1.16–1.24). The weight-average molecular weights (M_w_) of HTM 442, HTM 334, and HTM 226 were 32.4, 36.8, and 28.5 kDa, respectively. Although some variation in molecular weight was observed among the three compositions, the polymers remained within a similar molecular-weight range and exhibited relatively narrow molecular-weight distributions. No pronounced high-molecular-weight fraction was observed in the major GPC distributions, suggesting that extensive intermolecular coupling was not dominant under the employed polymerization conditions.

**Figure 4:**
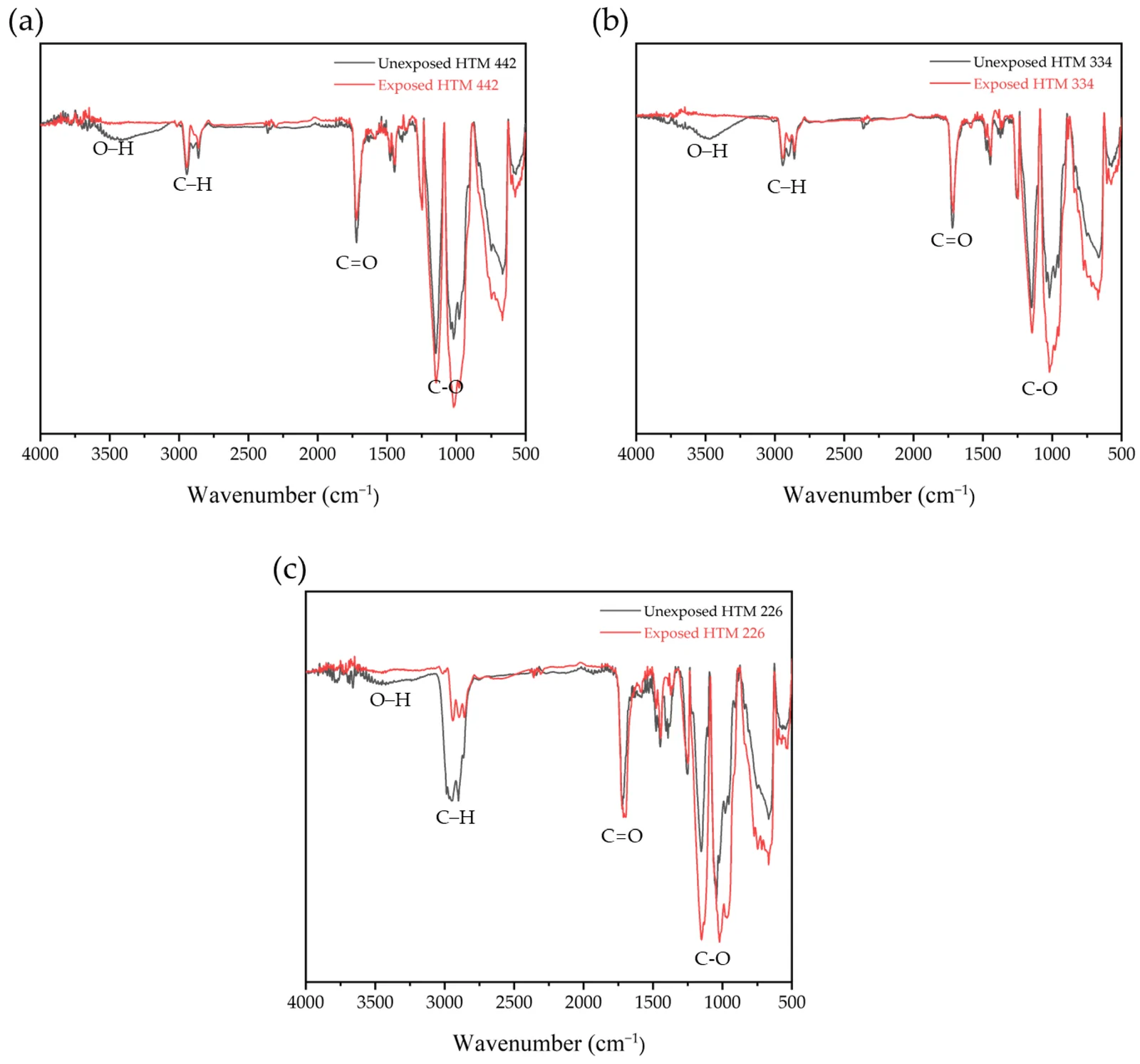
FT-IR spectra of the synthesized 3-arm star terpolymers before and after UV exposure and post-exposure baking: (**a**) HTM 442, (**b**) HTM 334, and (**c**) HTM 226.

The chemical functionalities of the synthesized polymers were additionally evaluated by FT-IR spectroscopy (Figure 4). All three samples exhibited absorption bands associated with methacrylate-derived ester carbonyl and C–O functionalities, together with characteristic aliphatic C–H absorptions originating from the cycloaliphatic groups. These spectral features further support the incorporation of the intended functional units into the polymer structures.

To semi-quantitatively evaluate the spectral changes induced by UV exposure and post-exposure baking, the transmittance spectra were converted to absorbance, and the O–H stretching region at 3200–3600 cm^−1^ was integrated after identical baseline correction and normalized to the integrated C=O region at 1650–1800 cm^−1^. The *A*_O-H_/*A*_O=H_ values decreased from 0.511 to 0.129 for HTM 442, from 0.446 to 0.244 for HTM 334, and from 0.250 to 0.059 for HTM 226, corresponding to relative attenuations of approximately 74.8%, 45.2%, and 76.3%, respectively.

The decrease in the 3200–3600 cm^−1^ band should not be interpreted as a direct decrease in the total number of hydroxyl-containing functionalities or as a direct measure of MAMA deprotection efficiency. This region is dominated by the HEMA-derived alcoholic O–H contribution and is also strongly affected by hydrogen bonding and the local chemical environment. In contrast, acid-catalyzed cleavage of the 2-methyl-2-adamantyl ester generates carboxylic-acid functionality, whose strongly hydrogen-bonded O–H absorption is considerably broader and extends toward lower wavenumbers. Therefore, formation of carboxylic-acid groups does not necessarily produce an increase in the apparent 3200–3600 cm^−1^ band intensity. Accordingly, the observed O–H attenuation is interpreted as a relative exposure/PEB-induced spectral change rather than as a quantitative deprotection conversion.

Taken together, the ^1^H NMR and GPC results confirmed the preparation of three compositionally distinct 3-arm star terpolymers with relatively narrow molecular-weight distributions, while FT-IR spectroscopy further supported the presence of the intended functional groups and revealed exposure/PEB-induced changes in their local chemical environments. The NMR-determined compositions provide the basis for comparing the thermophysical and lithographic behavior of HTM 442, HTM 334, and HTM 226 within the investigated compositional series.

### 3.2. Effect of Copolymer Composition on Thermophysical Properties

**Figure 5:**
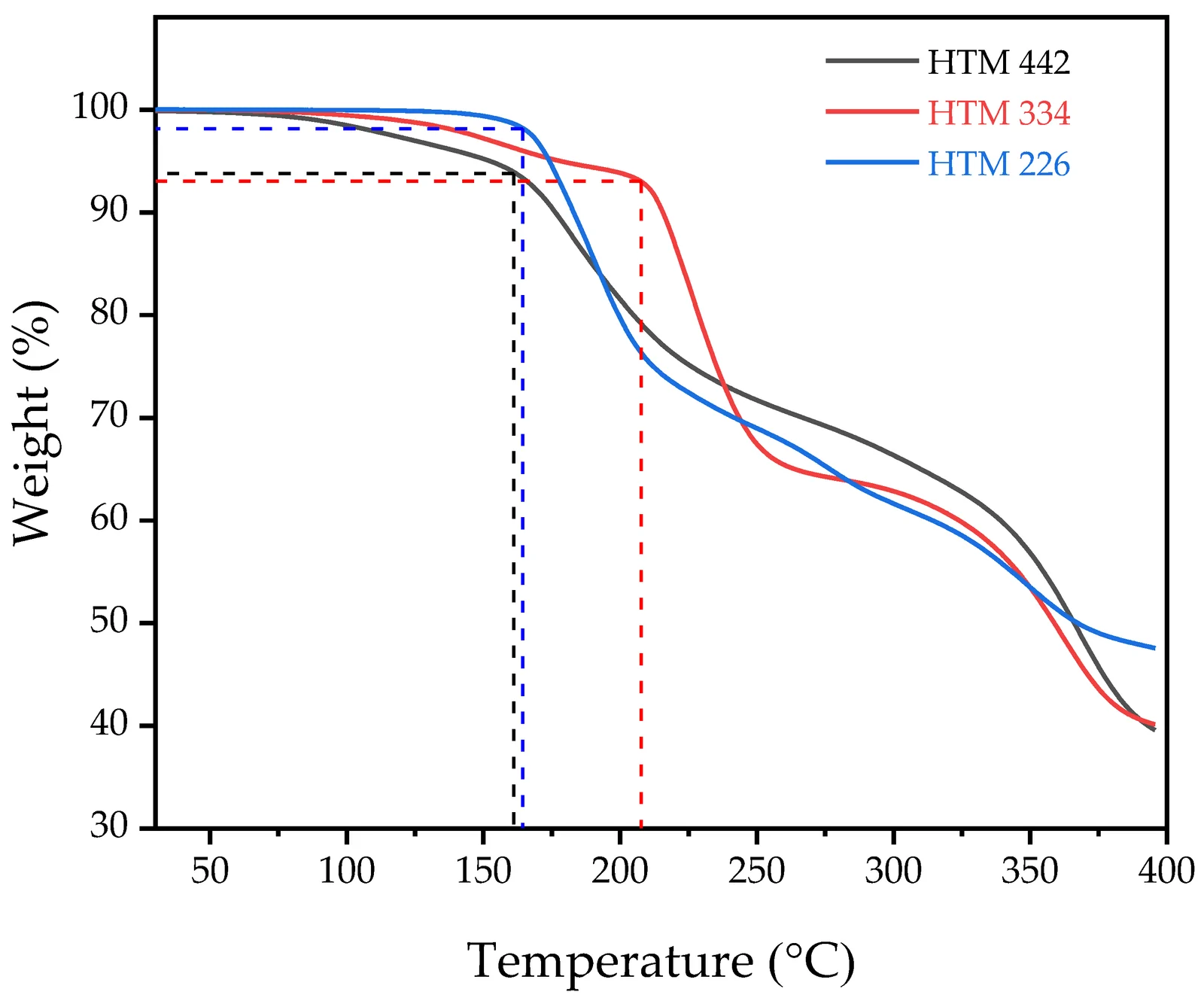
Thermal analysis of the synthesized 3-arm star terpolymers (the first-stage weight-loss events). The color-matched dashed lines serve as visual guides indicating the approximate first-stage weight-loss temperatures (HTM 442: 162 °C; HTM 334: 209 °C; HTM 226: 164 °C).

TGA revealed first-stage weight-loss events at approximately 162, 209, and 164 °C for HTM 442, HTM 334, and HTM 226, respectively. These initial thermal events were tentatively attributed to cleavage or degradation of acid-labile pendant groups, whereas the subsequent weight loss at higher temperatures was mainly associated with degradation of the polymer backbone (Figure 5). Among the three compositions, HTM 334 exhibited the highest first-stage weight-loss temperature. Importantly, this temperature did not vary monotonically with increasing MAMA content, indicating that the thermal response of the terpolymers cannot be interpreted solely in terms of the absolute MAMA fraction.

Differential scanning calorimetry (DSC) was performed using rigorously dried polymers under a heat–cool–heat protocol. No well-defined glass-transition step was observed within the investigated temperature range. Therefore, quantitative T_g_ values are not discussed in the present study, and the thermal characterization presented herein is based primarily on the TGA results.

### 3.3. Composition-Dependent Lithographic Performance

The lithographic performance of HTM 442, HTM 334, and HTM 226 was evaluated to investigate how the relative composition of HEMA, TCDMA, and MAMA influences the pattern-forming behavior of the 3-arm star photoresists. The three polymers were formulated as chemically amplified resists under identical processing conditions, allowing their lithographic behavior to be compared primarily in terms of copolymer composition. As confirmed by the structural analyses described above, all three polymers contained the acid-labile MAMA functionality required for the exposure-induced solubility transition.

**Figure 6:**
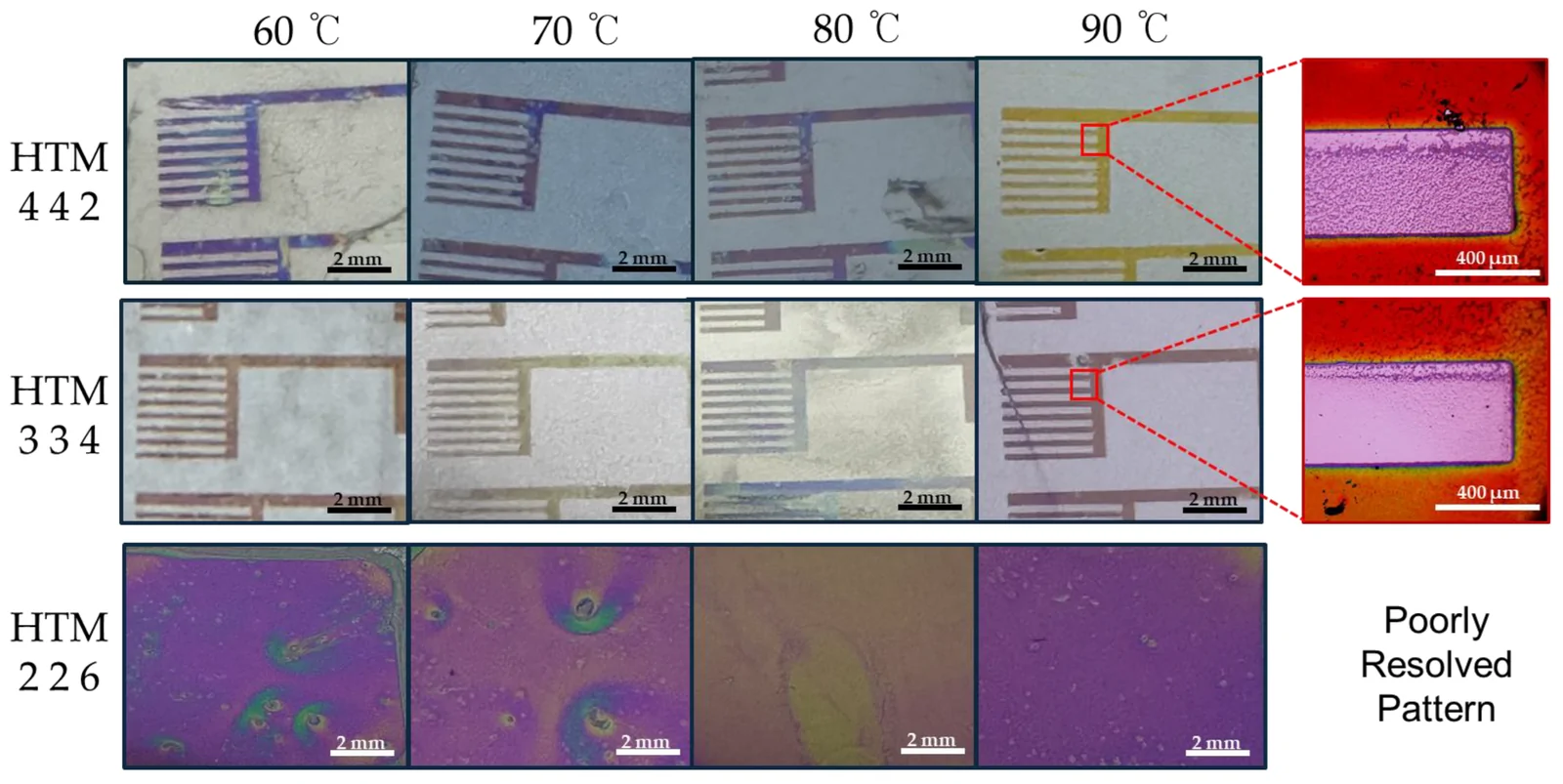
Optical microscopy images of negative-tone patterns obtained from HTM 442, HTM 334, and HTM 226 after post-exposure baking at 60, 70, 80, and 90 °C for 1 min. Red boxes and dashed lines indicate the magnified regions shown on the right.

Distinct differences in pattern formation were observed among the three compositions, as shown in Figure 6. HTM 334 produced the most clearly defined and uniform patterns over the investigated processing conditions. HTM 442 also exhibited pattern formation; however, the resulting features showed greater deformation and non-uniformity, particularly at the selected condition. In contrast, HTM 226 exhibited substantially poorer pattern formation, indicating that increasing the fraction of the acid-labile MAMA unit did not directly translate into improved lithographic performance.

This result is particularly important because HTM 442 contains the lowest MAMA fraction among the three compositions. MAMA provides the acid-labile functionality responsible for the polarity and solubility change during the exposure and post-exposure process; therefore, a larger MAMA fraction could in principle increase the number of sites available for deprotection. However, the present patterning results indicate that the amount of acid-labile functionality alone is not sufficient to determine the overall resist performance. Instead, the present results demonstrate a non-monotonic dependence of pattern formation on MAMA content within the investigated composition range.

Despite having the lowest MAMA fraction among the three samples, HTM 442 still produced distinguishable negative-tone patterns, indicating that a relatively low MAMA fraction can provide sufficient exposure-responsive functionality under the investigated conditions. By comparison, increasing the MAMA fraction in HTM 334 and particularly in HTM 226 simultaneously decreases the relative fractions of HEMA and TCDMA. Consequently, the chemical and physical environment surrounding the acid-labile groups is altered, which may affect deprotection, polymer–developer interactions, and the uniformity of dissolution during development. In contrast, HTM 334, which contains an intermediate MAMA fraction among the three samples, exhibited the most favorable pattern morphology under the investigated processing conditions. However, the present dataset does not permit assignment of this behavior to a uniquely defined optimum HEMA/TCDMA/MAMA balance.

The poorer patterning behavior of HTM 226 may therefore originate from an imbalance between its highly hydrophobic, bulky MAMA-rich protected state and the dissolution behavior required after exposure. Although a high concentration of MAMA provides a larger potential population of acid-responsive groups, the increased fraction of bulky adamantyl moieties may also alter local packing, chain mobility, and polymer–developer interactions and dissolution behavior in *n*-butyl acetate. These factors could lead to less uniform development even when substantial deprotection occurs. Further quantitative dissolution analysis would be required to distinguish the individual contributions of these effects; therefore, the present results are more appropriately interpreted in terms of an overall composition-dependent response rather than a single MAMA-content effect.

Taken together, the lithographic evaluation demonstrates that increasing the fraction of the acid-labile protecting group does not necessarily improve pattern quality in the present 3-arm star resist system. HTM 334 exhibited the most favorable lithographic response among the three investigated compositions, while the MAMA-rich HTM 226 showed poor pattern formation. These results demonstrate a non-monotonic dependence of lithographic behavior on MAMA content within the investigated composition range, without establishing a uniquely optimized HEMA/TCDMA/MAMA composition.

**Figure 7:**
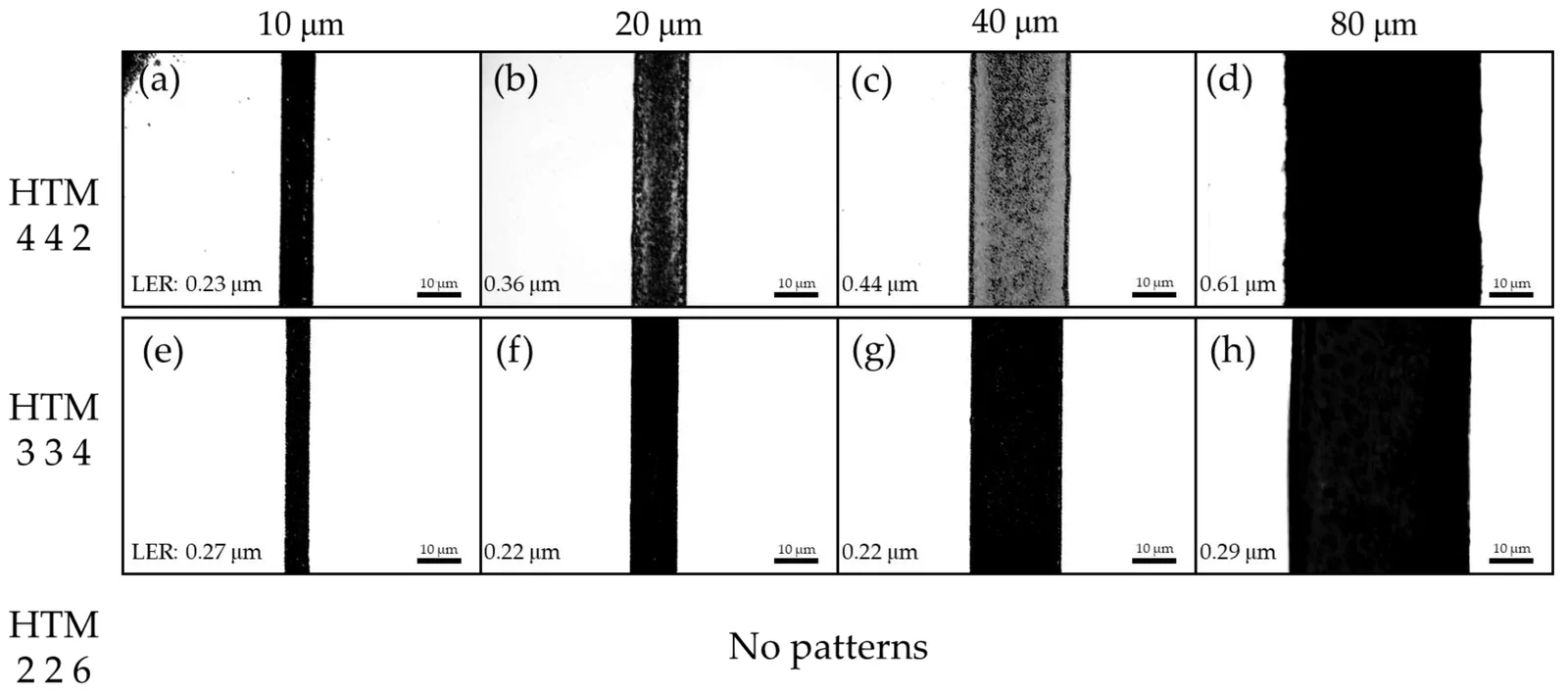
Optical microscope (OM) images of line patterns formed at nominal mask dimensions of 10, 20, 40, and 80 μm after PEB at 90 °C for 1 min: (**a**–**d**) HTM 442 and (**e**–**h**) HTM 334. HTM 226 did not produce distinguishable line patterns under the investigated conditions.

To further evaluate the pattern-forming behavior of the synthesized resists, line patterns with nominal mask dimensions of 10, 20, 40, and 80 μm were formed under the selected PEB conditions and examined by optical microscopy (Figure 7). Both HTM 442 and HTM 334 successfully generated distinguishable line patterns across the investigated mask dimensions. However, HTM 334 exhibited relatively clean and uniform line profiles, whereas HTM 442 showed greater line deformation and edge irregularity, particularly at larger nominal mask dimensions. This difference is consistent with the measured LER values, which were lower for HTM 334 than for HTM 442 at 20, 40, and 80 μm. In contrast, HTM 226 did not produce distinguishable line patterns under the evaluated conditions. These results further support the composition-dependent patterning behavior shown in Figure 6 and confirm that increasing the MAMA fraction alone does not produce a monotonic improvement in negative-tone pattern formation within the investigated series.

## 4. Conclusions

In this study, a series of 3-arm star terpolymers with different HEMA, TCDMA, and MAMA compositions were synthesized via core-first RAFT polymerization using the trifunctional TMP-CEPA3 chain-transfer agent. HTM 442, HTM 334, and HTM 226 were prepared with systematically varied MAMA fractions, and their structural, thermal, and lithographic properties were investigated. The synthesized polymers exhibited distinct copolymer compositions with relatively narrow molecular-weight distributions. Thermal analysis revealed that the thermal behavior of the star terpolymers did not depend solely on the MAMA fraction, but was influenced by the combined effects of all functional monomer components. Negative-tone lithographic evaluation demonstrated clear composition-dependent differences in pattern formation. HTM 334 exhibited the most favorable line-pattern morphology, showing relatively uniform line profiles and lower LER values than HTM 442 at most of the investigated nominal mask dimensions. HTM 442 also exhibited distinguishable pattern formation, whereas MAMA-rich HTM 226 showed poor pattern formation under the investigated conditions. The results show that increasing the acid-labile MAMA content alone does not produce a monotonic improvement in lithographic performance within the investigated series. HTM 334 exhibited the most favorable response among the three compositions examined; however, the present three-point dataset does not establish a quantitative composition–property relationship or a global optimum composition. A more densely sampled compositional series will be required to define the optimum compositional window quantitatively.

## Figures and Tables

**Table 1:** Nominal monomer feed ratios, ^1^H NMR-determined copolymer compositions, weight-average molecular weights (M_w_), and dispersities (Đ) of HTM 442, HTM 334, and HTM 226.

Polymer	Monomer Feed Ratio (HEMA:TCDMA:MAMA)	Copolymer Composition ^1^H NMR (HEMA:TCDMA:MAMA)	M_w_ (kDa)	PDI (*Ð*)
HTM 442	4:4:2	3.0:4.4:3.1	32.4	1.21
HTM 334	3:3:4	3.0:3.3:4.0	36.8	1.16
HTM 226	2:2:6	2.0:1.8:5.7	28.5	1.24

Note: The sample codes HTM 442, HTM 334, and HTM 226 refer to the nominal HEMA:TCDMA:MAMA monomer feed ratios.

**Table 2:** Estimated molecular characteristics related to acid-responsive functionality.

Polymer	Apparent Mn (kDa)	Estimated DP	MAMA Fraction (mol%)	Estimated MAMA Units per Star Polymer
HTM 442	26.8	128	29.5	38
HTM 334	31.7	153	38.8	59
HTM 226	23.0	103	60.0	62

Note: Apparent M_n_ values were estimated from M_w_/Đ. Estimated MAMA units were calculated using the NMR-determined copolymer compositions and composition-weighted repeat-unit molecular weights. These values should be regarded as approximate molecular-level estimates because the molecular weights were determined by GPC using relative calibration.

## Data Availability

The original contributions presented in the study are included in the article; further inquiries can be directed to the corresponding author.
